# The BACH1/Nrf2 Axis in Brain in Down Syndrome and Transition to Alzheimer Disease-Like Neuropathology and Dementia

**DOI:** 10.3390/antiox9090779

**Published:** 2020-08-21

**Authors:** Marzia Perluigi, Antonella Tramutola, Sara Pagnotta, Eugenio Barone, D. Allan Butterfield

**Affiliations:** 1 Department of Biochemical Sciences, Sapienza University of Rome, 00185 Rome, Italy; antonella.tramutola@uniroma1.it (A.T.); sara.pagnotta@uniroma1.it (S.P.); eugenio.barone@uniroma1.it (E.B.); 2 Department of Chemistry, University of Kentucky, Lexington, KY 40506, USA; 3 Sanders-Brown Center on Aging, University of Kentucky, Lexington, KY 40536, USA

**Keywords:** oxidative stress, BACH1, Nrf2, Down syndrome, Alzheimer disease

## Abstract

Down syndrome (DS) is the most common genetic cause of intellectual disability that is associated with an increased risk to develop early-onset Alzheimer-like dementia (AD). The brain neuropathological features include alteration of redox homeostasis, mitochondrial deficits, inflammation, accumulation of both amyloid beta-peptide oligomers and senile plaques, as well as aggregated hyperphosphorylated tau protein-containing neurofibrillary tangles, among others. It is worth mentioning that some of the triplicated genes encoded are likely to cause increased oxidative stress (OS) conditions that are also associated with reduced cellular responses. Published studies from our laboratories propose that increased oxidative damage occurs early in life in DS population and contributes to age-dependent neurodegeneration. This is the result of damaged, oxidized proteins that belong to degradative systems, antioxidant defense system, neuronal trafficking. and energy metabolism. This review focuses on a key element that regulates redox homeostasis, the transcription factor Nrf2, which is negatively regulated by BACH1, encoded on chromosome 21. The role of the Nrf2/BACH1 axis in DS is under investigation, and the effects of triplicated BACH1 on the transcriptional regulation of Nrf2 are still unknown. In this review, we discuss the physiological relevance of BACH1/Nrf2 signaling in the brain and how the dysfunction of this system affects the redox homeostasis in DS neurons and how this axis may contribute to the transition of DS into DS with AD neuropathology and dementia. Further, some of the evidence collected in AD regarding the potential contribution of BACH1 to neurodegeneration in DS are also discussed.

## 1. Genetics of Oxidative Stress in Down Syndrome

Down syndrome (DS) or trisomy 21 is one of the most common genetic disorder displaying phenotypic features that include neurodevelopmental defects, neuronal dysfunction, and accelerated aging, among others Cenini, Dowling [1,2,3]. Further, DS individuals are at increased risk to develop a type of dementia that mimics the clinical and pathological features course of Alzheimer disease (AD), with the deposition of amyloid plaques and neurofibrillary tangles. Interestingly, DS is emerging as a disorder etiologically related to oxidative stress (OS) mainly due to triplication of Cu, Zn-superoxide dismutase (SOD-1), encoded on chromosome 21 (Hsa21). However, recent reports showed that OS is driven not only from overexpression of some *Hsa21* genes, but also from a dysregulation of gene/protein expression associated with the trisomy [4].

OS results from either elevated production of reactive oxygen and nitrogen species (ROS/RNS) or by reduced antioxidant responses. The central nervous system (CNS) contains high levels of fatty acids that in the presence of high metabolic flux are a fertile ground for lipid peroxidation reactions responsible of generating increasing amount of free radicals as well as highly reactive products, such as 4-hydroxynonenal (HNE) [5,6,7,8]. In addition, superoxide anion (O_2_^•−^), hydrogen peroxide (H_2_O_2_), and hydroxyl radical (HO^•^), are continuously produced as by-products of aerobic respiration and various other catabolic/anabolic processes [9]. As a result, neuronal cells are highly susceptible to redox imbalance and to accumulate oxidative damage [10].

As an essential link to OS, mitochondrial dysfunction occurs whenever redox imbalances overcome the intracellular defense system, due to the main role of mitochondrial activity in oxygen metabolism and ROS production [11]. By-products of normal mitochondrial metabolism and homeostasis include the build-up of potentially damaging levels of ROS. A major source of free radicals is the mitochondrial oxidative phosphorylation pathway (OXOPHOS), in which electron leakage causes the formation of O_2_^•−^ that, in turn, is converted by mitochondrial resident manganese superoxide dismutase (MnSOD) into H_2_O_2_ and O_2_ [12,13]. In line with this evidence, dysfunction of complex I has been found to be involved in the overproduction of ROS in skin fibroblast from DS, isolated from both fetal and adult subjects [14].

Accumulating studies implicate OS in DS pathological phenotypes [15,16,17], though the exact mechanisms through which oxidative damage translate into clinical features of DS need to be clarified. It is likely that OS is a chronic condition in DS brain, that initiates already during embryonic development and further accumulates with aging, representing a strong risk factor for subsequent neurodegeneration [18,19].

In order to better understand and appreciate the causes of OS in DS brain, initial explanations can be obtained by mapping the Hsa21 on which a number of genes (Table 1) such as SOD1, amyloid precursor protein (APP), the transcription factor BTB and CNC homology 1 (BACH1), the Protein C-ets-2 (ETS2), carbonyl reductase (CBR), S100 calcium-binding protein B (S100B), among others, are directly involved in the overproduction of ROS as found in DS individuals and in mouse models thereof [20].

Among trisomic genes, *SOD1* is one of the first lines of antioxidant defense by catalyzing the conversion of O_2_^•−^ to molecular oxygen (O_2_) and H_2_O_2_, which can be neutralized by catalase (CAT) and by glutathione peroxidase (GPX) to water [21]. Moreover, the triplication of Hsa21 is not accompanied by a parallel increase of CAT and GPX, resulting in imbalance in SOD1/CAT levels and those of SOD1/GPX, with an accumulation of H_2_O_2_ [22]. Interestingly, in all DS tissues an altered SOD-1/GPX activity ratio has been observed [5], that may partially explain higher levels of H_2_O_2_ and its by-products. In addition to CAT and GPX, a decreased expression of peroxiredoxin 2 also was detected in DS fetal brain, which further contribute to the increased susceptibility of DS neurons to undergo oxidative damage [23].

Intriguingly, aberrant expression of SOD seems to be associated with mitochondrial impairment. Indeed, transgenic mice overexpressing wild-type human SOD1 (Tg-SOD1) show many mitochondrial defects such as increased mitochondrial swelling and vacuolization, that also are associated with learning and memory disturbance [7]. In addition, Tg-SOD1 mice have altered levels of ATP synthase alpha/beta chain and elongation factor Tu, while no changes in the levels of antioxidant proteins were observed [7]. Taken together, these alterations correlate with synaptosomal damage and neuronal loss in the brain of Tg-SOD1, ultimately leading to cognitive deficits in DS.

Several studies reported that defects of mitochondrial structure and function are associated with increased ROS production [24,25,26,27]. In line with this assumption, mitochondrial dysfunction is considered a pathological signature of DS, also in the presence of increased OS condition [25]. Neurons of DS patients exhibited a significant increase in intracellular ROS levels together with elevated lipid peroxidation [5,26,27]. The DS mitochondrial phenotype includes: (i) reduced ability to produce ATP through OXPHOS; (ii) decreased respiratory capacity; and (iii) disruption of mitochondrial membrane potential, all events associated with loss of mitochondrial dynamics. These mitochondrial defects are present in all DS cell types, from peripheral tissues to the brain [28].

In addition to the well-recognized role of SOD1, increased OS could also be caused by the over-production of Aβ, due to triplication of APP. This hypothesis is confirmed by a number of reports demonstrating that both Aβ (1-40/42) are able to induce OS, as in the case of AD [8,29]. Indeed, Butterfield and others [8,16,30,31] proposed that Aβ (1-42), in the form of oligomers, is able to insert into the membranes initiating lipid peroxidation and downstream cascades [29]. Accordingly, the levels of both Aβ (1-42) and Aβ (1-40) in plasma are higher in DS compared with non-DS controls and deposition of senile plaques is observed in post-mortem brain from DS individuals [32,33], very early in life. Further, Aβ has the ability to coordinate metal ions—Zn^2+^, Cu^2+^ and Fe^2+^—and the alteration of metal homeostasis is known to regulate both production and defense against ROS and is also involved in the regulation of neuronal activity in the synapses and other biological functions in the brain. It is interesting to underlie that studies from Anandatheerthavarada et al. [34] for the first time provided evidence that full length APP itself is able to damage mitochondria. Consistent with their work, mice overexpressing wild type human APP show cognitive defects and neuronal pathology similar to what observed in AD models, though these mice do not show significant Aβ deposition in the hippocampus [35]. These findings support the notion that, in addition to effects from APP-generated Aβ oligomers on mitochondria, trisomy of APP itself may promote mitochondrial dysfunction in DS.

BACH1, encoded on Hsa21, is a key element in the regulation of the antioxidant response in DS [6]. BACH1 is a transcription repressor that acts as a key regulator of the expression of genes involved in the cell stress response [36]. In DS, it is likely that upregulation of BACH1 protein levels could block the induction of antioxidant genes, therefore promoting increased OS in the cell [6]. The molecular aspects of BACH1 triplication will be discussed in the next section.

By mapping Hsa21, another candidate gene that is related to OS is the enzyme carbonyl reductase (*CBR*). CBRs are NADPH-dependent cytosolic enzymes with broad substrate specificity for many endogenous and xenobiotic carbonyl compounds. They catalyze the reduction of endogenous prostaglandins, steroids, and other aliphatic aldehydes and ketones. Carbonyls are considered toxic metabolic intermediates, that can be detoxified both through oxidation by aldehyde dehydrogenase (ALDH) or by CBR-mediated reduction and/or alcohol dehydrogenase (ADH). Increased levels of both these enzymes were detected in the brain of both DS and AD patients, likely in response to elevated carbonyls production [37].

Published studies from our laboratories identified several oxidatively modified proteins in DS brain, prior and after development of AD [5,38,39]. Among several targets, oxidation of proteins could be particularly deleterious in aging and in age-related neurodegenerative diseases, due to a gradual loss of efficiency of clearance systems for their removal [37]. The role of OS in the development and progression of AD in the general population has been extensively discussed in several review papers [8,40,41]. Among the proteins identified by redox proteomics to be oxidatively modified, either by increased carbonylation or HNE modification, proteins involved in several intracellular processes such as (i) neuronal trafficking; (ii) the proteostasis network; (iii) energy metabolism; and (iv) mitochondrial function were found [6].

Reduced ATP levels, increased ROS, and altered mitochondrial permeability are characteristic mitochondrial defects of degenerating neurons not only in DS but also in many neurodegenerative disorders, including AD [8]. Overall, proteomics data demonstrate that oxidative damage is an early event in DS, and the dysfunction of protein clearance systems contributes to increased neuronal vulnerability to oxidative damage that accelerate neurodegenerative phenomena. As noted, considering that several of the above-mentioned proteins have been already found to be oxidized in AD brain, our results strongly support the notion that aberrant protein oxidation in DS may contribute to age-dependent AD risk.

This view is also confirmed by a longitudinal study analyzing some redox markers in plasma samples from DS subjects (1–57 years old) showing that changes in redox-related parameters are strongly age-dependent [17]. Similarly, Pallardo and colleagues showed a significant increase in 8-hydroxy-2-deoxyguanisine (8-OHdG) levels in DS patients up to 30 years old. Levels of antioxidants including ascorbic acid and vitamin E also were evaluated, showing that plasma concentrations of ascorbic acid are increased in young (<15 years) DS individuals, but not in older persons, while the levels of vitamin E did not differ from control either in young or older DS individuals compared with non-DS healthy individuals [17].

The picture that emerges from both brain and peripheral studies suggest that young DS individuals are characterized by an early pro-oxidant state [27] that results in a variety of pathological phenotypes. With age, adult DS persons accumulate oxidative damage associated with an increased risk to develop Alzheimer-like dementia [20,22]. Understanding the complexity of factors regulating redox homeostasis may help to identify potential therapeutic treatments able to prevent the accumulation of oxidative damage. In this scenario, it is particularly interesting to discuss the role played by BACH1/Nrf2 axis in DS.

## 2. BACH1/Nrf2 Signaling

Based on the considerations above, it is conceivable that OS occurs in DS pathogenesis and progression due to a dysregulation of gene/protein expression associated with the trisomy. As noted above, OS represents an imbalance between the production of ROS and the ability of a biological system to detoxify the reactive toxic intermediates—the antioxidant response—or to repair the resulting damage.

A prominent sensor involved in the antioxidant response is the Keap1-Nrf2-ARE (Kelch-like ECH-Associating protein 1- nuclear factor erythroid related factor 2-antioxidant response element) signaling complex. The transcription factor nuclear factor erythroid 2-related factor 2 (Nrf2) mediates induction of multiple antioxidant enzymes through activation of ARE of DNA. Activation of Nrf2 results in cellular protection by increasing the expression of antioxidant enzymes such as NADPH quinone oxidoreductase 1 (NQO1), heme oxygenase 1 (HO-1), and multiple components of the glutathione pathway. Promotion of this production of antioxidants is therefore a promising mechanism to protect against neurodegeneration. Under normal OS conditions, Keap1, a cysteine-rich protein that senses redox changes in the cell, binds to Nrf2 leading to retention of Nrf2 in the cytosol and causing its proteasomal degradation [42,43]. Under OS, conformational changes in Keap1 lead to its dissociation from the Nrf2-Keap1 complex and to the translocation of free Nrf2 into the nucleus, where it binds to ARE regions in the genome, to activate the expression of stress response genes [44]. So far, there is no complete information on Nrf2/Keap1 genes, protein levels or activities in DS. However, a recent study comparing gene expression profiles in DS and euploid astrocytes found that Nrf-2-associated oxidative stress response genes were differentially regulated in DS [45]. In addition, a study from Swatton et al. in DS reported that mitogen-activated protein kinases (MAPKs) are highly phosphorylate in DS and AD brains [46], and this result can be linked to the mechanism whereby MAPKs phosphorylate Nrf2 enabling its dissociation from the Nrf2/Keap1 complex, but preventing its translocation into the nucleus.

Compared to Nrf2, ARE transcriptional repressors and their roles in DS, AD, and other neurodegenerative disorders have been minimally investigated. Indeed, a novel hypothesis regards the implication of BACH1 to compete with the Keap1-Nrf2-ARE signaling complex. BACH1 is a member of the Cap “n.” Collar and basic region leucine zipper family (CNC-bZip) of transcription factors is encoded on HSA21 and functions primarily as a transcriptional repressor. Human BACH1 is composed by 736 amino acids: (a) N-terminal region of BACH1 contains a BTB/POZ domain, which functions as a protein interaction motif; (b) while the C-terminal bZip domain binds to DNA forming heterodimers with small Maf proteins (i.e., MafK, MafF, and MafG) [47]. Once into the nucleus, BACH1-Maf heterodimers are able to inhibit the transcription of many oxidative stress-response genes. In addition, BACH1 contains six cysteine-proline (CP) motifs, four of which are located in a heme-binding region near the C- terminus. Heme is able to inactivate BACH1 by interacting with two of the CP motifs, leading to the exclusion of BACH1 from the nucleus [48]. Under pro-oxidant condition, nuclear BACH1 binds heme, changes its conformation, dissociates from ARE and allows transcription factors to bind and activate the expression of oxidative stress-responsive genes [49].

The export of BACH1 from the nucleus is also the result of its tyrosine phosphorylation, activated by the antioxidant response, (BACH1 tyrosine 486) [50] and by cadmium, which induces a cytoplasmic localization signal in the BACH1 C-terminus [51]. After its release in the cytoplasm, BACH1 forms fiber-like structures on microtubules in the presence of intracellular hyaluronic acid-binding protein (IHABP), which regulates the subcellular localization of the former [51].

Many of the genes targeted by BACH1 are in common with the genes regulated by Nfr2 and participate in redox regulation, including HO-1, that is crucial for cell survival upon detrimental oxidative stress conditions. HO-1 expression is negatively regulated by BACH1 when heme levels are reduced, though higher heme levels are able to inhibit BACH1-DNA interaction and also promote BACH1 nuclear export and its subsequent degradation [52]. This event induces HO-1 expression, which in turn degrades heme while releasing antioxidant molecules such as carbon monoxide (CO), and biliverdin. Thus, the BACH1/HO-1 pathway is considered to act as a feedback loop that regulates heme homeostasis during sustained oxidative stress.

**Figure 1:**
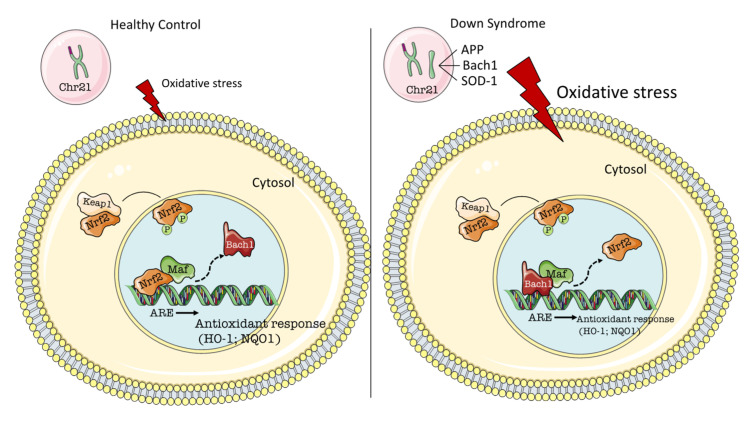
**BACH1/Nrf2 signaling regulates antioxidant response**. Under normal conditions (**left**), Keap1/Nrf2 retains Nrf2 in the cytoplasm. Exposure of cells to oxidative insult leads to the release of Nrf2 from Keap1/Nrf2. Nrf2 then translocates to the nucleus and binds to MAREs as a heterodimer with small Mafs, thereby activating oxidative stress-response genes (e.g., HO-1 and NQO1), while Bach1 is displaced from MAREs and exported out of the nucleus. In trisomic cells (DS) (**right**), triplication of BACH1 competes with Nrf2 thus resulting in reduced binding of Nrf2 to MAREs. This event may explain the increased oxidative stress levels of DS individuals, that is likely the result of compromised induction of antioxidant response.

Taken together, BACH1 is believed to displace Nrf2 from AREs [49] and to act primarily as a transcriptional repressor for antioxidant genes, such as HO-1 [53] and NQO1 [54]. Specifically, BACH1 competes with Nrf2 for binding to the AREs in oxidative stress-response genes. In response to OS, Nrf2 dissociates from Keap1, translocates into the nucleus, and binds to AREs as a heterodimer with Mafs, thereby activating oxidative stress-response genes as previously described, while BACH1 is displaced from AREs and exported out of the nucleus [49] (Figure 1). A recent study suggests that both the nuclear import of Nrf2 and the dissociation of BACH1-ARE are promoted by sirtuin-6 (Sirt 6) [55]. Research from Dhakshinamoorthy et al. demonstrated that positive and negative regulation of ARE-mediated gene expression depend on the critical balance between Nrf2 and BACH1 in the nucleus. This was clearly evident from the observation that BACH1 repression of ARE-mediated gene expression was relieved by co-expression of Nrf2 with BACH1, and BACH1 failed to repress the ARE in cells overexpressing Nrf2. However, BACH1 repressed the ARE activation in cells expressing moderate levels of Nrf2 [54].

In light on these findings, focusing on DS individuals, the triplication of genes (e.g., *BACH1*) encoded on HSA21 is directly involved in the appearance of harmful conditions such as increased OS, which we hypothesize over time contributes to the early development of AD pathology in DS individuals. The presence of BACH1 on HSA21 opens the possibility of seeking new therapies capable of controlling the possible imbalance between Nrf2 and BACH1 in the nucleus.

## 3. Involvement of BACH1 in AD and DS

The evaluation of BACH1 functions in the brain and particularly in neurodegenerative disorders characterized by a failure of antioxidant responses, represents a novel aspect of DS research. Indeed, only a limited number of studies addressed this topic to date.

Studies performed in BACH1 knock-out mice (BACH1^−/−^) showed significantly higher HO-1 mRNA expression levels with respect to control animals in all brain regions studied [56]. Moreover, higher induction of HO-1 was observed around damaged tissues in BACH1^−/−^ mice [56]. Similar results were collected with regard to spinal cord where HO-1 protein levels were found significantly higher in BACH1^−/−^ than WT mice either before or after injury [57]. Furthermore, neuronal loss and apoptotic cell death in the injured spinal cord was significantly reduced in BACH1^−/−^mice [57]. Thus, as described above, these results confirm that BACH1 plays an important role in regulating HO-1 expression levels in the central nervous system.

The first evidence about a possible involvement of BACH1 in brain disorders came in 2003 when Shim et al. evaluated levels of BACH1 protein levels in a small cohort of post-mortem frontal cortex samples collected from DS, AD, and related control individuals to test the hypothesis that DS-phenotype may be due to the overexpression of genes encoded on chromosome 21 [58]. Despite the prevalent hypothesis of a gene-dosage effect in DS, these authors found that BACH1 protein levels were significantly reduced in DS samples, suggesting that DS features cannot be simply explained by the overexpression of triplicated genes [58]. However, these findings were revised by the same group published in a subsequent study [59]. In the afore-mentioned work, fetal cortical specimens from DS fetuses and controls (females) from the 18–19th week of gestation were used to evaluate BACH1 protein levels and levels of one of the targets of BACH1 activity, i.e., HO-1, among the others [59]. BACH1 was significantly overexpressed in fetal DS as compared to controls [59], while the levels of HO-1 were found comparable between the two groups [59]. In light of these results these researchers concluded that increased BACH1 did not lead to decreased HO-1, which would have explained oxidative stress observed in fetal DS [59].

However, we suggest that the above conclusion could be criticized. Considering that HO-1 is inducible, with lower intracellular levels under physiological conditions, HO-1 levels are dynamically responsive to a variety of oxidative and inflammatory stimuli such as heme, Aβ, H_2_O_2_, heavy metals, UV light, hyperoxia, prostaglandins, nitric oxide (NO), peroxynitrite, lipopolysaccharide, oxidized lipid products and various growth factors [60,61,62]. Further, in the adult brain, HO-1 expression in basal conditions is restricted to sparse clusters of neurons and glia [60]. While in the unstressed rodent brain, low-level of HO-1 is observed in scattered neurons of the cerebral cortex, hippocampal dentate gyrus, thalamus, hypothalamus, and cerebellum [63,64,65,66,67]. Hence, reduced HO1 levels in DS frontal cortex might not be expected. The reason could simply be that increased BACH1 levels would prevent HO1 overexpression, which remains at similar levels to those observed in the control group. If considered in this way, no changes observed for HO-1 levels would lead to increased oxidative stress levels in DS brain, since HO-1 is among the first proteins induced to elicit an antioxidant response under conditions of increased oxidative stress levels [68,69,70].

This hypothesis was further strengthened by proposing a role for BACH1 overexpression as one of the causes driving the development of neurodegeneration in DS [6]. Indeed, the expression levels and the ubiquitinylation of BACH1 were evaluated in post-mortem frontal cortical samples isolated from postmortem DS persons before and after (DSAD) the development of AD neuropathology, compared to age-matched controls [6]. Moreover, the incident effects of BACH1 on HO-1 and on its physiological partner biliverdin reductase-A (BVR-A)—both involved in the production of the antioxidant molecule bilirubin [60,61]—as well as the levels of NQO1, were determined. Results from this study highlighted that BACH1 protein levels are significantly elevated in DS subjects, either before or after the development of AD [6]. Furthermore, the evaluation of BACH1 post-translational modifications revealed that BACH1 mono-ubiquitinylation levels were reduced only in DS, while increased levels of BACH1 poly-ubiquitinylation were observed only in DSAD subjects [6].

In parallel, it was observed that neither HO-1 or NQO1 protein levels (both regulated by BACH1, [53,54]) were different between DS and age-matched controls, while they were significantly increased in brains from the DSAD group [6]. Observations collected from DS individuals are in agreement with those previously reported by the group of Lubec and co-workers [59]. Moreover, further studies contributed to extending the knowledge about the regulation of BACH1 in DS. Indeed, by taking into consideration that mono-ubiquitinylation is involved in modulating protein function, compartmentalization, and interactions, while polyubiquitinylation is a signal for protein degradation [71,72], collectively, the results suggest that more than the expression levels, the regulation of BACH1 activity/degradation plays a role in DS and contributes to the explanation of observed changes with regard to HO-1 [6]. Increased BACH1 protein levels along with reduced BACH1 mono-ubiquitinylation would be responsible for the lack of HO-1 or NQO1 increase observed in DS, while increased BACH1 poly-ubiquitinylation (degradation) would drive the observed increase of HO1 and NQO1 protein levels in DSAD persons [6]. Increased BACH1 poly-ubiquitinylation could result from increased oxidative stress levels in DSAD individuals [6]. Similar analyses performed in a mouse model of DS, i.e., Ts65Dn mice, at different ages further suggest that BACH1 overexpression results from the triplication of chromosome 21, although the mechanisms associated with BACH1 regulation appear different between mice and humans [6]. Indeed, the overexpression of BACH1 was not associated with differences in the ubiquitinylation profile between Ts65Dn and control mice [6]. Increased BACH1 levels in Ts65Dn mice tend to maintain HO-1 expression levels comparable to those of euploid animals [6].

The pivotal role for BACH1 in the regulation of antioxidant response in DS is further highlighted by data collected with regard to BVR-A. As indicated above, BVR-A is normally co-expressed with HO-1 to promote the degradation of pro-oxidant heme into the antioxidant molecule bilirubin [61,73]. Notwithstanding that BVR-A possesses pleiotropic functions through which this enzyme regulates intracellular signaling [73]. Among these functions, Maines’ group identified BVR-A as a heme-binding and heme transport protein, and suggested its role in the modulation of the expression of heme-regulated genes [74]. In particular, these researchers proposed that transport of heme to the nucleus by BVR-A would enable its delivery to the transcriptional repressor BACH1, which, on binding heme dissociates from the DNA, and is replaced by the Nrf2 transcription factor [74]. Indeed, cells lacking BVR-A were characterized by reduced HO-1 expression in response to heme [74]. Moreover, increased BVR-A levels were observed in both DS and DSAD individuals with respect to age-matched controls [6]. However, they were not associated with increased HO1, particularly in DS, further suggesting that over-expression of BACH1 precludes HO-1 upregulation mediated by physiological activators, including BVR-A.

Studies about the possible involvement of BACH1 in AD neuropathology mostly rely with in vitro analyses. The only report aimed to evaluated levels of BACH1 in AD brain shows no changes with respect to control subjects [58]. This gap in knowledge about functions of BACH1 in AD needs to be fulfilled in future studies, particularly in light of the role for HO1 and reduced antioxidant response in the onset and progression of AD pathology [60,61,75]. While HO-1 and NQO1 protein proteins levels are elevated in AD brain (reviewed in [61,76]), reduced Nrf2 activity was reported in a number of studies performed on human and animal samples. Indeed, reduced Nrf2 nuclear expression in hippocampal samples from AD subjects were observed [77]. Similarly, a failure of Nrf2-mediated processes in AD mouse models was reported [78,79,80,81]. Together, these observations spur the necessity for deeper investigations into a possible role for BACH1 in AD.

A previous study showed that BACH1 also targets the gene for microtubule-associated protein tau (also known as MAPT)—known to drive AD progression [82]—by repressing its expression [83]. In this context, Koglsberger et al. reported that BACH1 expression contributes to molecular gender differences observed in tauopathies and AD and provides a new target for intervention strategies to modulate MAPT expression [84]. Since gender differences may influence the risk for brain disorders and the severity of their phenotypic manifestations, the role for BACH1 appears of great interest. Indeed, there is evidence of a gender difference in the phenotypic expression of AD in DS. Female middle-aged DS individuals have an earlier onset and a more severe form of AD that correlates with higher neocortical neurofibrillary tangles (NFT) rather than senile plaques (SP) density (2).

Among the in vitro studies that assessed a role for BACH1 in neuronal injury, Piras et al. evaluated the effects of increased oxidative stress levels, mimicked by H_2_O_2_, in differentiated SH-SY5Y cells [85]. A striking finding of this work is represented by the fact that in differentiated cells BACH1 is not displaced from the HO-1 promoter and Nrf2 is not allowed to bind, while maintaining its ability to sense H_2_O_2_ moving into the nucleus [85]. In addition, BACH1 and Nrf2 mRNA levels were not modified by increased oxidative stress levels, further corroborating the hypothesis that the main regulation of both BACH1 and Nrf2 occurs at the post-transcriptional level [85]. These results agree with findings from our group with regard to DS (cited above) and reinforce the hypothesis that the sole evaluation of protein and/or transcripts levels are not sufficient to unravel the molecular mechanisms regulated by BACH1 (Table 2).

Finally, a role for specific micro RNAs (miRNAs) in regulating BACH1 expression was identified. miRNAs are key regulatory molecules, given their ability to disrupt expression of target mRNAs [86]. With the attempt to better clarify potential regulators of BACH1 in DS, Tili et al. evaluated the expression levels of miR155, which is one of the miRNA encoded on chromosome 21 that also targets the BACH1 gene [87]. These scientists found that miR-155 is overexpressed only in DS brain, but not in control samples or even in AD samples took as reference [87]. Furthermore, these researchers found BACH 1 protein was strongly expressed in the DS fetal brain tissues, but not in adult DS with dementia with respect to controls [87]. Reduced BACH1 levels in DS with dementia with respect to AD samples were also observed [87]. miR-155/BACH1 levels strongly correlated with the disease process in DS [87]. The results from Tili et al. are apparently in contrast to the overexpression of BACH1 found by Di Domenico et al. [6]. However, there are two key points that deserve attention because they represent differences between the two studies. The number of DS samples (samples were collected from 3 DS donors in Tili et al. [87] vs. 8 samples in Di Domenico et al. [6]), and the age of the donors was different as well (34 to 42 in Tili et al. [87] vs. 59 in Di Domenico et al. [6]). Furthermore, in Tili et al. AD samples were not matched for age with Control or DS, were used [87]. Hence, in our opinion, the conclusions about the strong association between miR-155/BACH1 and the development of dementia in DS require more extensive investigation.

Another miRNA identified through a bioinformatic analysis as a potential regulator of BACH1 is miR-98-5p [88]. miR-98-5p is a stress-related miRNA that plays an important role in regulating cell survival, apoptosis, and oxidative stress in multiple cell types and diseases [88]. Sun et al. reported that miR-98-5p alleviates neuronal injury induced by oxygen-glucose deprivation/reoxygenation, by also reducing reactive oxygen species levels [88]. Indeed, overexpression of miR-98-5p significantly suppressed the expression of BACH1 at the mRNA and protein levels, while inhibition of this micro RNA promoted the expression of BACH1 [88]. miR-98-5p overexpression also significantly increased the nuclear translocation of Nrf2 and promoted the activity of ARE [88]. As a consequence, the transcription of NQO1 and HO-1 also were significantly upregulated [88]. The role of miR-98-5p was confirmed by showing that BACH1 overexpression reversed the neuroprotective effects mediated by miR-98-5p overexpression [88]. These findings suggest a neuroprotective role for miR-98-5p by affecting the oxidative stress response through BACH1 suppression.

## 4. Concluding Remarks

Collected evidence suggests that young DS individuals are characterized by an early pro-oxidant state that contributes to a variety of pathological phenotypes. Among the genetic elements involved in the regulation of redox homeostasis, triplication of *SOD1* and *BACH1*, are considered to play a major role. Since SOD1 is directly responsible for production of hydrogen peroxide, triplication of BACH1 has wider effects because of its role in the BACH1/Nrf2 axis-induced antioxidant response. Considering that with age adult DS individuals accumulate oxidative damage in brain associated with an increased risk to develop Alzheimer-like dementia, modulation of the BACH1/Nrf2 axis in DS may represent a promising therapeutic strategy. Further studies are needed to dissect the complexity of targets that are controlled by BACH1/Nrf2 signaling and the potential mechanisms that are relevant to aging and neurodegeneration not only in DS but also in AD and, potentially, in other neurodegenerative disorders.

## Figures and Tables

**Table 1:** List of some genes located on Hsa21 linked to OS, as discussed in the text.

Gene on Hsa21	Molecular Function	Biological Process	Relevance in Down Syndrome
**SOD-1** (Cu,Zn-superoxide dismutase 1)	Oxidoreductase	Antioxidant Response	Triplication of SOD-1 in DS brain results in an imbalance in the ratio of SOD-1 to CAT and GPX (two enzymes involved in its metabolism), thus leading to the accumulation of H_2_O_2_ in the cells.
**APP** (amyloid precursor protein)	Heparin-binding, Protease inhibitor	Apoptosis, Cell adhesion, Endocytosis, Notch signaling pathway and Aβ processing	Triplication of APP causes the over-production of Aβ (1-40/42) in DS brain. Deposition of senile plaques of Aβ is observed in post-mortem brain and plasma from DS compared with non-DS individuals.
**BACH1** (*BTB Domain and CNC Homolog 1)*	DNA-binding, Transcription regulation	Antioxidant Response	Triplication of BACH1, as a negative transcription regulator, in DS brain could block the induction of antioxidant genes, therefore promoting increased OS in the cell.
***CBR*** *(Carbonyl reductase)*	Oxidoreductase	Oxidative stress Response	Triplication of CBR in DS play a role in exacerbating OS. Carbonyls are toxic metabolic intermediates that are mainly detoxified by aldehyde dehydrogenase or reduced by CBR and/or alcohol dehydrogenase to their corresponding alcohols. Increased levels of these enzymes were detected in the brain of DS patients, likely in response to elevated carbonyls production in DS.
***ETS2*** *(Protein C-ets-2)*	Transcription regulation	Cell differentiation, maturation and signaling	Triplication of Ets-2 could play a role in the increased susceptibility of DS cells to undergo apoptosis given common pathophysiological features shared between Ets-2 overexpressing transgenic mice and individuals with DS.
***S100B*** *(S100 calcium-binding protein B)*	Ca^2+^-binding protein	Neurotrophic factor	Triplication of S100β in DS corresponds to an increase of its expression levels in astrocytes in association with neuritic plaques. In addition, chronic overexpression of S100β contributes to increased neuronal and neuritic βAPP expression with consequent accelerated amyloid deposition, as well as abnormal growth of neurites in β-amyloid plaques, similar to observations in middle-aged DS patients.

**Table 2:** Summary of BACH-1 alterations observed in Down syndrome and Alzheimer disease.

Pathology	BACH1 Changes	References
**Down Syndrome (DS)**	↑ BACH1 protein levels in fetal cortical specimens of human DS	Ferrando-Miguel R.J. Neural Transm Suppl, 2003(67): p. 193-205 Tili, E., et al., Ann Diagn Pathol, 2018. 34: p. 103-109.
	↑ BACH1 protein levels in human DS subjects, either before or after the development of AD	Di Domenico, F., et al., J.Alzheimers Dis, 2015. 44(4): p. 1107–20.
	Changes in post-translational modifications of BACH-1:	Di Domenico, F., et al., J.Alzheimers Dis, 2015. 44(4): p. 1107–20.
	↓ mono-ubiquitination of BACH1 in young DS human brain	
	↑ poly-ubiquitinylation of BACH1 only in DSAD subjects	
	↑ BACH1 protein levels in brain of Ts65Dn mice.	Di Domenico, F., et al., J.Alzheimers Dis, 2015. 44(4): p. 1107–20.
	No changes were observed in BACH1 ubiquitination in Ts65Dn mice compared to euploid mice.	
**Alzheimer Disease (AD)**	NO changes were observed in BACH1 protein levels in AD brain.	Shim, K.S., R. Ferrando-Miguel, and G. Lubec, J Neural Transm Suppl, 2003(67): p. 39–49.
	↑ BACH1 protein levels in AD Brain using an immunohistochemistry approach.	Tili, E., et al., Ann Diagn Pathol, 2018. 34: p. 103–109.

## Abbreviations

Aβ	amyloid beta-peptide
AD	Alzheimer disease
APP	amyloid precursor protein
ARE	antioxidant response element
BACH1	the transcription factor BTB and CNC homology 1
BVR-A	biliverdin reductase A
CAT	catalase
CBR	carbonyl reductase
CO	carbon monoxide
DS	Down syndrome
DSAD	Down syndrome with Alzheimer disease
ETS2	the Protein C-ets-2
HNE	4-hydroxy-2-nonenal
HO-1	heme oxygenase 1
HSA21	human chromosome 21
Keap1	Kelch-like ECH-Associating protein 1
MAPK	mitogen-activated protein kinase
MAPT	microtubule associated protein tau
NFT	neurofibrillary tangles
NO	nitric oxide
NQO1	NADPH quinone oxidoreductase 1
Nrf2	nuclear factor erythroid 2 related factor 2
OXOPHOS	oxidative phosphorylation
OS	oxidative stress
ROS	reactive oxygen species
SOD1	Cu, Zn superoxide dismutase 1
MnSOD	manganese superoxide dismutase
SP	senile plaques
S100B	S100 calcium-binding protein B
